# The role of plasma volume and fluid overload in the tolerance to ultrafiltration and hypotension in hemodialysis patients

**DOI:** 10.1080/0886022X.2022.2151917

**Published:** 2023-01-12

**Authors:** Jesús Manolo Ramos-Gordillo, Carlos Pérez-Campuzano, Elizabeth Relles-Andrade, José Carlos Peña-Rodríguez

**Affiliations:** Centro de Diagnóstico Ángeles (CEDIASA), Mexico City, México D.F.

**Keywords:** Ultrafiltration, dry weight, fluid overload, plasma volume hypotension, water spaces, bio-impedance

## Abstract

**Introduction:**

Ultrafiltration (UF) in hemodialysis (HD) patients is accompanied by irregular falls in plasma volume (PV) and blood pressure (BP).

**Methods:**

We obtained in 321 patients (large cohort), body weight (BW), BP, samples of blood to determine hemoglobin (Hb) and hematocrit (Ht), Pre and Post HD. We estimated the % variation of the PV and its effect on the BP. In a small cohort of 38/321 patients, arterial blood was drawn Pre and Post HD and at 2, 48, and 72 h to determined Hb and Ht and % variation of the PV. Bio-impedance spectroscopy (BIS) was performed, in the same times, to estimate: dry weight (DW), total body water (TBW), extracellular water (ECW), Fluid overload (FO) and phase angle (PhA).

**Results:**

We divided our large cohort in two groups. The Hypotensive group with a fall equal or more than 20 mmHg (96/321,30%) and Normotensive group with a drop equal or less than 19 mmHg (225/321,70%). The UF was 2.73 ± 0.72 L in the Hypotensive group and 2.53 ± 0.85 L in the Normotensive group (*p* < 0.0001). The % PV was −11.7 ± 17.8 in the Hypotensive group and −8.53 ± 10.07 in the Normotensive group (*p* < 0.0001). The systolic blood pressure (SBP) correlated with the % change of the PV (*r* = -0.232; *p* < 0.0001). The FO was contrasted with the % of water removed by UF (*r* = -0.890; *p* < 0.0001).

**Conclusion:**

The SBP drop was secondary to the fall in the PV after UF. The FO was irregular and modulates in part the fall in the SBP.

## Introduction

The decrease in plasma volume (PV) after ultrafiltration (UF) is followed by a fall in intra and post dialytic blood pressure (BP) in, 12–20% of hemodialysis (HD) patients. This decrease is not necessarily proportional to the fall in the PV. Different factors have been considered to explain this discrepancy [1–5]. One of the factors proposed, in the speed of recovery of the PV and the fall in the blood pressure (BP), was the overhydration or fluid overload (FO) [3,6]. The rate of refilling of the PV, regulates the decrease in this space and the tendency to develop significant falls in BP [2]. According to Bellizzi et al. [7] the recovery of the PV occurred in the first two hours after UF [8]. This behavior of the PV was not confirmed in other studies [9] or with our results. One of the factors in the irregular refill of the PV, might be the FO or overhydration. Presently the dilemma of this irregular individual behavior of the PV remains elusive [3,4,9]. Part of the problem rests, in our incapability to determine with precision, the dry weight (DW). Bio-impedance spectroscopy (BIS) is a method utilized to estimate more accurately the DW and the FO [10–13].

In this study, we evaluate the role played of UF in the BP, the PV, and the body water spaces; and the influence of the FO on these variables during and after HD [2–13].

The decrease in the PV after UF stimulates hemodynamic and neuro-hormonal factors, which may persist for several hours, particularly in subjects with different degree of FO and without renal function, and may contribute, to episodes of hypotension [2–14].

## Methods

### Subjects

**Figure 1.**
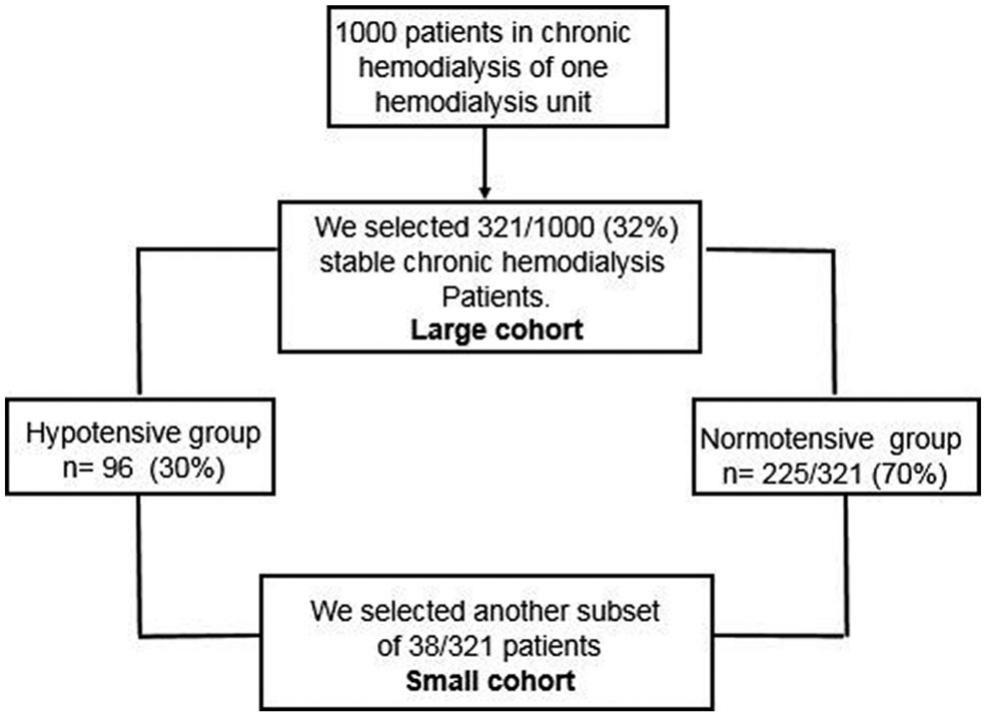
Flow chart of the 321/1000 patients selected from our patients in the chronic hemodialysis program.

We selected a large cohort 321/1000 patient from one of our Hemodialysis Units. (See Flow chart, Figure 1). In them, we obtained BP before, during and after HD; blood samples before and after hemodialysis. Hypotension was defined as a fall ≥10 mmHg in the mean arterial pressure (MAP), and ≥20 mmHg in the systolic BP (SBP), according to KDOQI clinical definition of 2005 and other definitions [15,16]. We divided the patients into two groups. The Hypotensive group with a fall equal or more than 20 mmHg (96/321, 30%) and Normotensive group with a drop equal or less than 19 mmHg (225/321, 70%). We also divide our patients in those with SBP Pre HD< 140 mmHg 121/321(38%) and those with SBP >140 mmHg 200/321(62%); and analyze the impact of the SBP Post HD. In all our patients hypotensive drugs were suspended 48–72 h before the study. The patients within the normotensive group with SBP below 130 mmHg did not receive hypotensive drugs.

Prospectively we selected another subset of 38/321 patient, with the propensity score matching (Figure 1). Hb and Ht was measured Pre, Post and at 2, 48, and 72 h after HD. At the same time points, we performed BIS.

This study was approved by the Ethical Local Committee of Centro de Diagnóstico Angeles, and all patients provided written inform consent to participate.

### Weight and bioelectrical impedance spectroscopy (BIS) description

To utilize the Seca medical composition model body 525, we needed body weight obtained with a measuring station platform Seca model 614, accuracy of ± 100 g, maximum weight 360 Kg. We estimated size with a combination of a scale and a stadiometer, accuracy ±0.5 cm; abdominal circumference with a non-stretchable measuring tape. We obtained bio-impedance in the supine position. A measuring mat is located above the knees, from there emerges four pairs of electrodes, that are positioned in each hand (wrist and hand) and in each foot (ankle and above the toes). Results are transmitted to the monitor via WIFI. We performed BIS with a current of 100 µA. Measurements take 50 s. With these values we obtained: Total body water (TBW), extracellular water (ECW), dry weight (DW), and muscular mass (MM). We estimated FO, subtracting DW from the Pre HD weight (Pre BW). We obtained the Phase Angle (PhA) at 50 kHz [17,18]. We compared our results against a reference standard of a Healthy Adult Latin-American population of both sexes. We included the data in the software of the Seca model mBSA 525. One of us (CPC) performed the BIS. The clinical characteristics of the 321 are displayed in Table 1.

**Table 1.** Demographics of the large cohort.

Variable	Data
Number of patients	321
Age (years)	59 ± 14
Female	137 (43%)
Male	184 (57%)
Diabetes	178 (55%)
Height (cm)	160 ± 10
Weight (Kg)	66 ± 15
Body mass index	25 ± 5
Body surface area (m^2^)	1.6 ± 0.5
Months in HD	72 ± 2

The etiology of renal disease overall was: Diabetes mellitus 185 (58%); Hypertension 38(12%); Glomerulonephritis 15(5%); Polycystic kidney disease 12 (4%); Others 33(10%); and Unknown 38 (12%).

### Hemodialysis procedure

All patients were dialyzed using Fresenius Machines 4008S, three times per week, for 3–4 h, with high flow, high efficiency and permeability with polysulphone dialyzers. We recorded body weight pre and post HD; blood flow (Qb), dialysis fluid flow (Qd) 500–600 mL/min, Temp 35.5 °C, Na 137–138 mEq/L; K 2.5 mEq/L and Calcium 2.5 mEq/L, UF indication varied from 10–20 mL/Kg/h. BP was recorded before and after the HD and along the procedure. Water was obtained from an elaborated central delivery system, with reverse osmosis. Water purity followed the standards of the AAMI, <200 colony forming units (CFU)/ml and <0.2 UI/ml of endotoxins.

### Blood chemistries

We obtained blood samples before and after HD, from the arterial line and measured, Hb and Ht in the same blood samples that, estimate Kt/V. We utilized low Qb (≤100 mL/min) for the recollection of the post HD samples. In the 38 patients sub-group, we recollected blood samples at, 2,48, and 72 h post HD. To estimate, Hb, Ht and mean concentration corpuscular Hb (MCCHb) with a Beckman Coulter model LH-750. All preHD blood samples included platelet and leukocyte count, glucose, urea, blood ureic nitrogen, serum creatinine, uric acid, total cholesterol, triglycerides, total protein, albumin, sodium, potassium, chloride, calcium, phosphorus and alkaline phosphatase (data not shown).

### Analysis and calculations

We obtained the blood volume (BV) in liters with Nader [19] formula:

(1a) $$\text{For}\text{men}=(0.3669\times {\left(\text{height}\;\text{in}\;\text{meters}\right)}^{3)}+\left(0.03219\times \text{weight}\;\text{in}\;\text{Kg}\right)+0.6041.$$

(1b) $$\text{For}\;\text{women}=\left(0.3561\times {\left(\text{height}\;\text{in}\;\text{meters}\right)}^{3}\right)\;+\left(0.3308\times \text{weight}\;\text{in}\;\text{Kg}\right)+0.1833.$$

Plasma volume (PV) as:

(2) PV=BV×(1−Ht)

Erythrocyte mass (EM) using the following equation:

(3) EM=BV–PV

Total Hb with the formula:

(4) Total Hb=EM×MCCHb

We use Ht and the formula described by Van Beaumont [20] to calculate the percent change of plasma volume (% PV):

(5) % △PV=100/(100−Ht Pre) ×100 (Ht Pre−Ht Post)/Ht Post).

A correction factor was introduced by Koomans [9] to estimate whole Ht (Venous Ht × 0.87). This correction factor included the so-called Fcells changes (the ratio of whole body to large vessel hematocrit) during circulatory stress, such as UF. Koomans [9] validated the formula described by Van Beaumont [20] using this correction factor and was compared with the gold standard using 131-labelled albumin. The results showed almost a perfect correlation (*r* = 0.98). With this background, we utilized with confidence the % PV estimated with Ht.

### Statistics methods

We used descriptive and inferential statistics in our data, and expressed values as mean ± SD, median or percentage, as appropriate. A *p* value <0.05 was considered statistically significant. For comparisons between groups, we used paired and non-paired *t* test or the variables assessed. We utilized the general linear model procedure with least–squares to identify significant interactions between groups and variables. We selected with the propensity score matching [21] another subset of 38 patients. The IBM SPSS STATISTICS 20 was used for all statistical calculations. In some analysis, we used the EXCEL 2013 statistics program.

## Results

### Large cohort results

**Figure 2.**
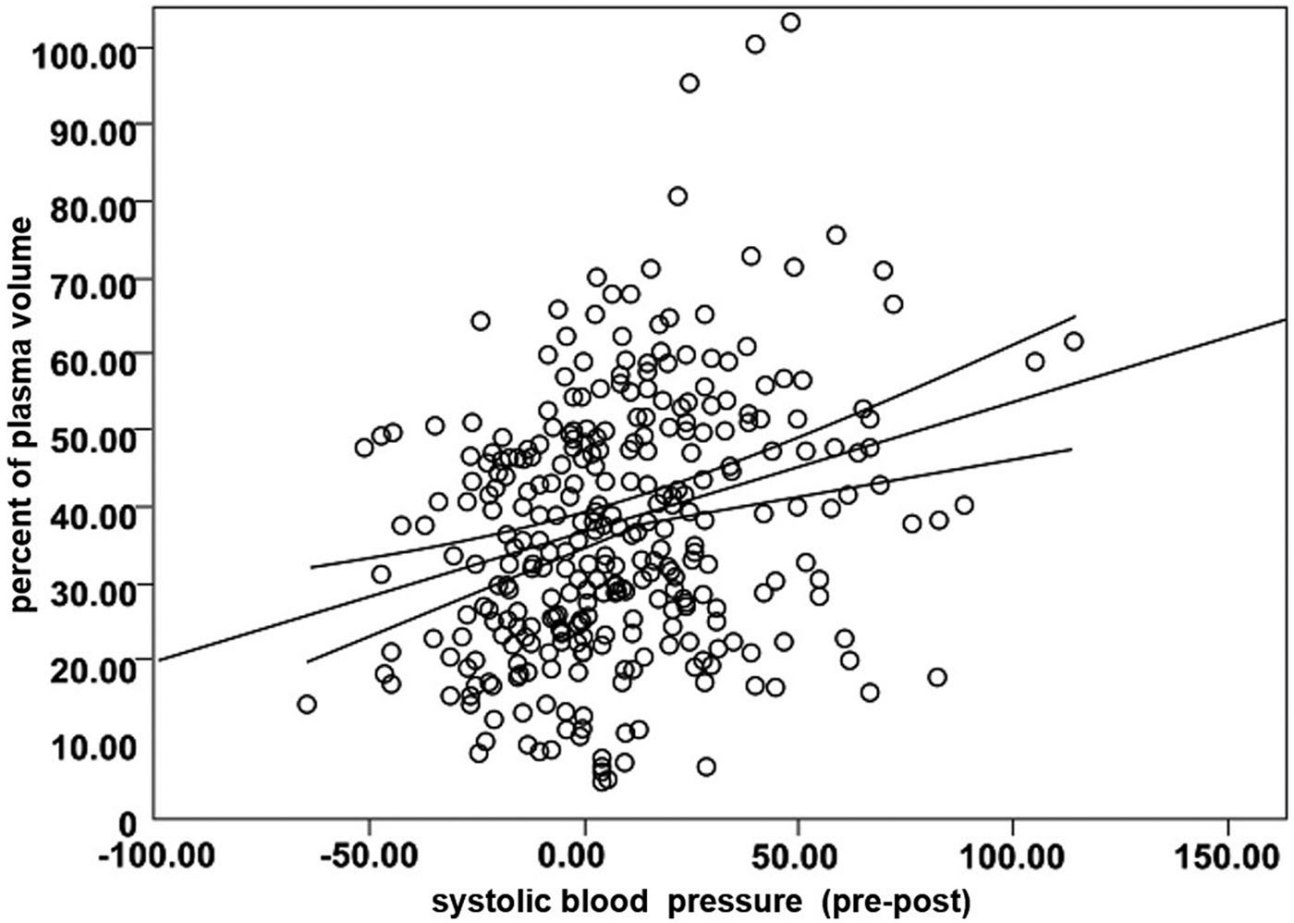
The figure shows a very significant negative correlation after UF, between the decreases in the systolic blood pressure vs the fall in percent of plasma volume, in 321 cases. (*r* = -0.232, *p* < 0.0001).

The clinical data obtained in all patients are shown in Table 1. In Table 2 are depicted: The fall of the BW before and after HD in Kg that was equal to the UF in liters. The reduction of the systolic BP (SBP), diastolic BP (DBP) and MAP after HD and UF, was very significant (*p* < 0.0001). We analyzed further that the decrease in the SBP, correlated with the % change of PV (Figure 2) (*r* = -0.232, *p* < 0.0001). The % PV vs. UF had a significant negative correlation (*r* = -0.384; *p* < 0.0001). The SBP and the MAP vs. UF showed a poor but significant correlation (*r* = 0.122; *p* < 0.023 and *r* = 0.179; *p* < 0.001).

**Table 2.** Results of the BP and the body weight before and after UF in the large cohort (*n* = 321).

Variable	Data
UF (L)	2.6 ± 0.82
Weight pre-post (Kg)	2.52 ± 1.12 NS
SBP pre HD (mmHg)	149.7 ± 28.4
SBP post HD (mmHg)	141.6 ± 28.6*
DBP pre HD (mmHg)	79.2 ± 12.8
DBP post HD (mmHg)	70.1 ± 15.8 *
MAP pre HD (mmHg)	102.8 ± 15.3
MAP post HD (mmHg)	93.8 ± 17.7 *

*Mean difference of BP pre and post HD; *p <* 0.0001.

NS mean difference UF vs weight pre-post HD NS.

In Table 3 we showed the results of the Hypotensive group with a significant decrease of the SBP of −40.3 ± 19 mmHg after HD; compared with the Normotensive group with a mean decrease of the SBP −8.8 ± 10.3; the table also shows that the SBP before and after HD in the Hypotensive group was significantly different from the Normotensive group (*p* < 0.0001). The UF was significantly higher in the Hypotensive than in the Normotensive group (*p* < 0.0001). In addition, the % PV fall was lower in the Hypotensive group than in the Normotensive group (*p* < 0.0001). The relationship between % PV vs UF in the Hypotensive group showed a very significant negative correlation (*r* =  −0.47 *p* < 0.0001) and confirms the findings in the 321 patients. Diabetes was more frequent in the Hypotensive group (77%), than in the Normotensive group 46%, the difference was significant (Table 3).

**Table 3.** Depicts the data of the large cohort divided in two groups those with a fall of ≤20 mmHg Normotensive group; and a drop of >20 mmHg, Hypotensive group; and the % of diabetic patients in both groups.

Variable	Normotensive group	Hypotensive group	*p*
Number of patients	225/321 (59.4%)	96/321 (40.6%)	
Diabetics	104/224 (46.4%)	74/96 (77.4%)	0.001
SBP pre mmHg	148 ± 28	167 ± 25	0.0001
SBP post mmHg	141 ± 26	126 ± 23	0.0001
SBP drop mm Hg	−8.3 ± 27.5	−40.3 ± 19.6	0.0001
UF liters	2.52 ± 0.85	2.73 ± 0.73	0.0001
%PV	−8.84 ± 10.4*	−11.56 ± 17.4	0.0001

We also divide our cohort, in patients with SBP above and below 140 mmHg Pre and Post HD. (Table 4). To analyze which group was more sensitive to develop hypotension. The mean fall of the SBP in the patients with SBP <140 mmHg after HD was 3.34 ± 20.56 mm Hg and below 20 mmHg we only detected 14/121 (11.4%) cases. In the group with SBP >140 the fall of the SBP was more pronounced −14.73 ± 27.8, and we found 82/200 (40%) patients with a decrease of <20 mmHg a significant difference with group with <140 mmHg (*p* < 0.0001). These results reflected that patients with SBP above 140 mmHg were more prone to severe hypotension.

**Table 4.** The 321 patients were divided in two groups SBP Pre HD ≤140 and ≥140 mmHg.

Variable	≤140 mmHg	≥140 mmHg	*p*
Number of patients	121/321 (38%)	200/321 (62%)	
SBP pre mmHg	121.4 ± 13.8	167.17 ± 19.7	0.0001
SBP post-pre mmHg	3.3 ± 20.5	−14.7 ± 27.8	0.0001
Cases with SBP fall <20 mmHg	14/121 (11.4%)	82/200 (40%)	0.0001

### Small cohort results

The Table 5 shows the clinical values obtained in the small cohort and the mean value of the UF of 2.6 ± 0.5 liters.

**Table 5.** Data of the small cohort (38 patients) obtained from the 321 patients with the propensity score matching.

Variable	Data
Number of patients	38
Age (years)	59 ± 14
Female	15 (43%)
Male	23 (57%)
Diabetes mellitus	16 (42%)
Body mass index	24.75 ± 4
Body surface area	1.7 ± 0.5
UF (L)	2.6 ± 0.55

Table 6 summarizes the results Pre and Post HD obtained with BIS plus the values of the FO, % PV, SBP and MAP. All values displayed decreased significantly after UF except DW and MAP. The FO in liters was directly proportional to the SBP Pre and Post HD (*r* = 0. 38; *p* < 0.02 and *r* = 0.54; *p* < 0.001). The PhA increased after HD, and correlated inversely with the amount of FO (*r* = -0.435; *p* < 0.007) and was directly proportional with the MM (*r* = 0.34; *p* < 0.049).

**Table 6.** Shows the results of the SBP, MAP and bioimpedance pre and post HD; plus the % PV and FO, in the small cohort.

*n* = 38	Pre HD	Post HD	*p*
Body weight (BW) Kg	65.5 ± 14	63.5 ± 14	0.01
Dry weight	62 ± 13	62 ± 13	NS
SBP	143 ± 29	135 ± 27	0.02
MAP	95 ± 18	91 ± 18	NS
UF in liters		2.6 ± 0.55	−−
TBW liters	36.4 ± 7	34.5 ± 7	<0.0001
ECW liters	17.4 ± 3	15.4 ± 3	<0.0001
PV in liters	2.8 ± 0.6	1.5 ± 2.2	<0.0001
FO liters	3.5 ± 2*	1.5 ± 2.2**	<0.001
Phase angle	4.6 ± 1	5.1 ± 2.2	<0.001

FO: Fluid overload; *(weight pre HD – dry weight) and **(weight post HD – dry weight).

**Figure 3.**
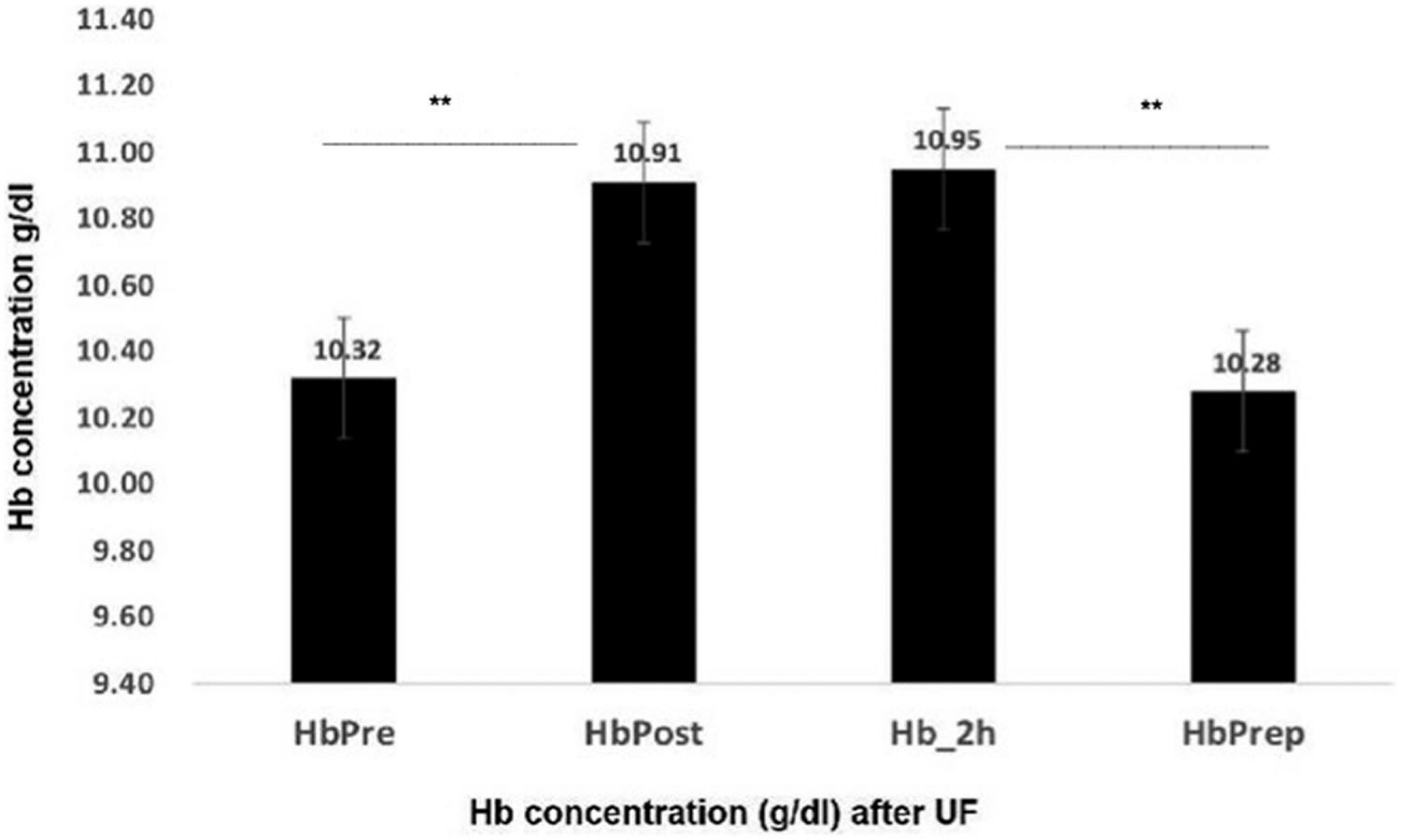
Change in Hb concentration after UF in the post and 2 h period in the small subgroup. Hb Prep (Hb 48/72 h). Differences between Hb Pre and Hb Prep (48/72 h) were NS. Hb Post and Hb 2 h were NS. Hb Pre and Prep were different from Hb Post and Hb 2 h (*p* < 0.001).

We obtained Hb and Ht before and after HD, and at 2, 48, and 72 h. We compared all values vs Hb Pre. The values of Hb Post and Hb at 2 h (Figure 3) were non-different (*p* < 0.7); but significantly different from Hb Pre (*p* < 0.001). The samples of Hb at 48 and 72 h were analyzed together and were equal to Hb Pre (Figure 3).

**Figure 4.**
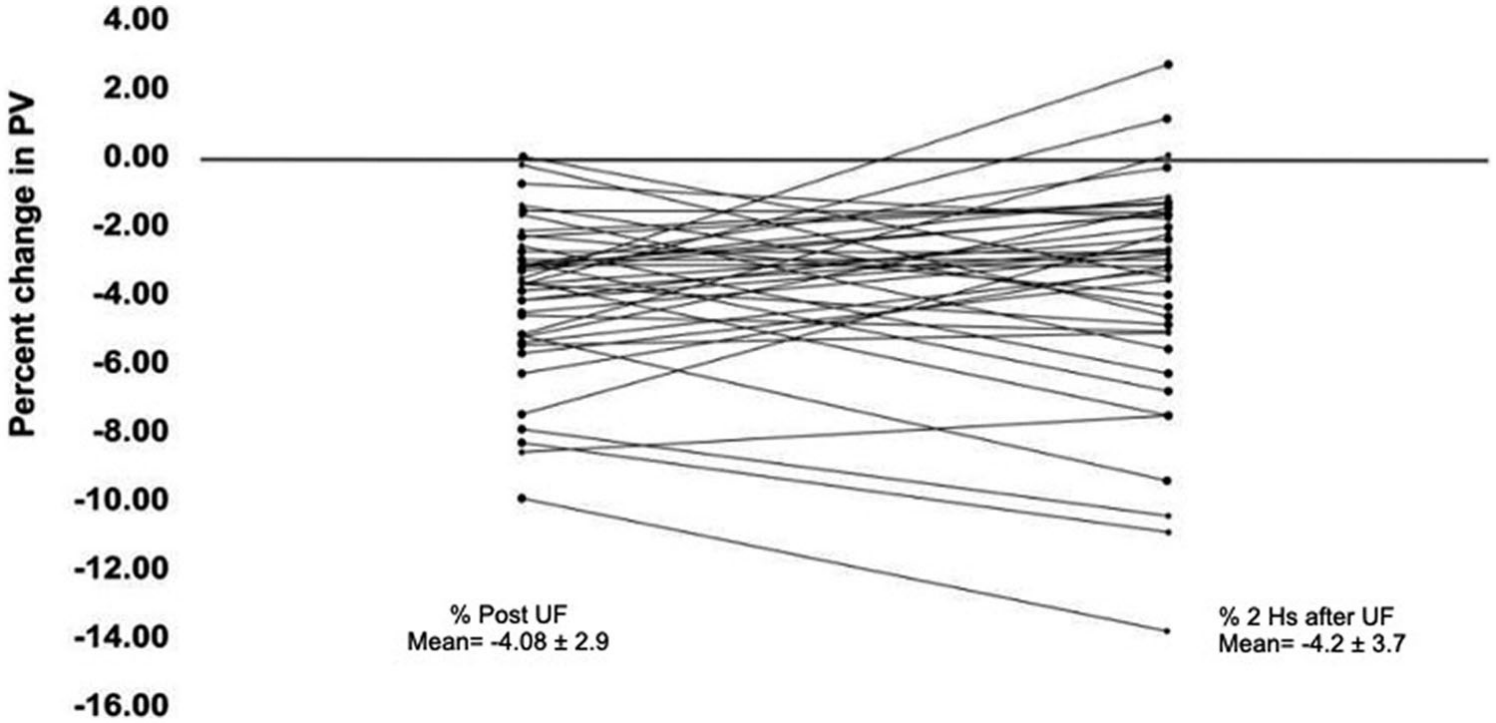
Recovery of percent of plasma volume, 2 hs after Uf, in the 38 cases studied (sub-group). Difference was NS.

To further look in the behavior of the recovery of the Ht Post and the Ht at 2 h we calculated the percent decrease of %PV after UF in the Post HD period and at 2 h; the results are displayed in (Figure 4). The refill of the PV at 2 h is incomplete and irregular and resulted in a family of curves. Only three cases of the 38 returned to control values. The mean value of the decrease of % PV Post UF was −4.08 ± 2.9 and at two hours − 4.2 ± 3.7; difference non-significant. This slow recovery of the PV may also contribute to the BP fall.

**Figure 5.**
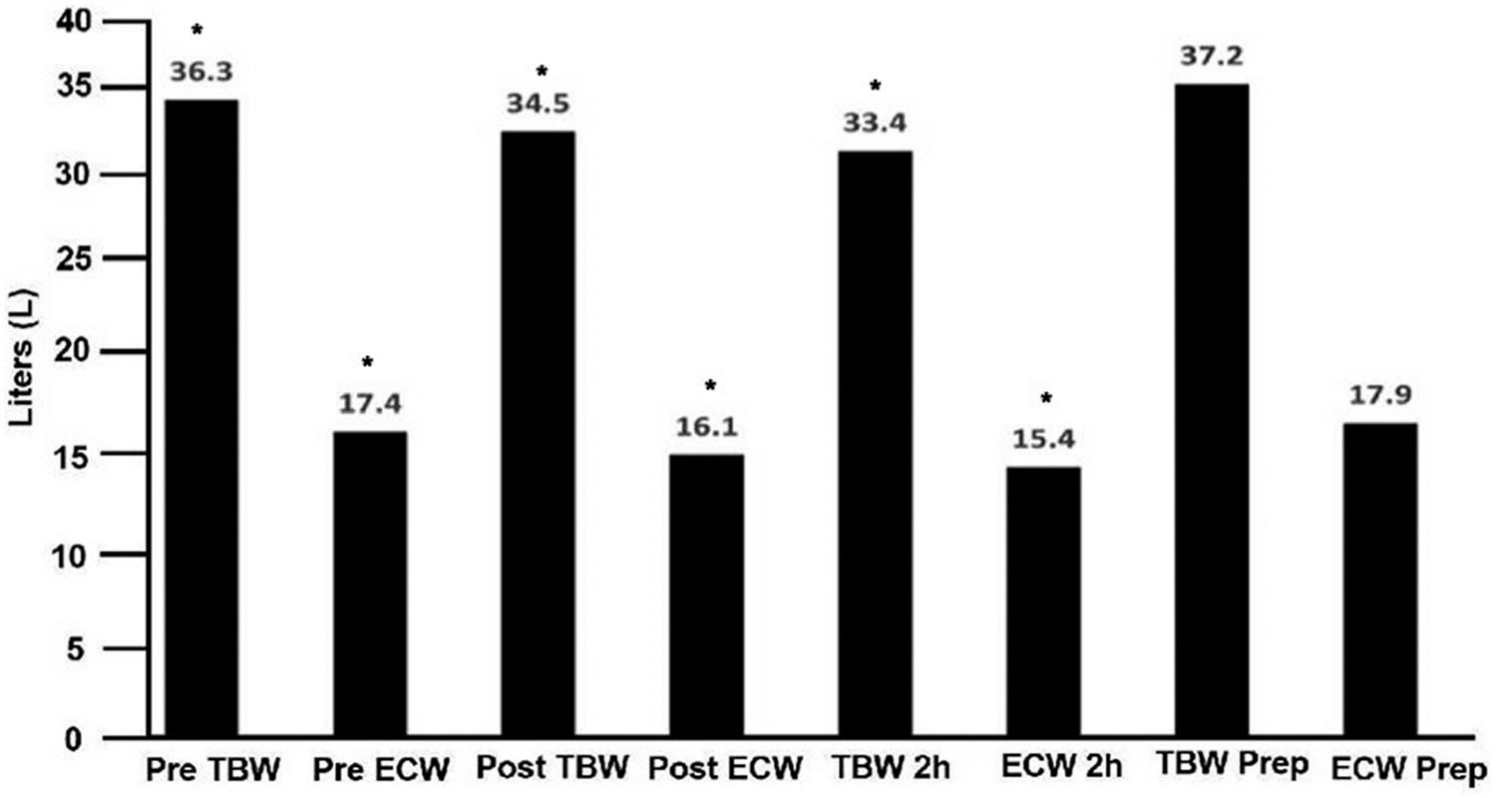
Pre TBW and pre ECW decrease significantly in post TBW and post ECW; TBW 2H and ECW, 2H TBW 2 h (*p* < 0.001). The recovery of TBW and ECW was complete at TBW Prep (48/72 hs) and ECW Prep. TBW Pre and ECW Pre were non different from TBW Prep and ECW Prep.

**Figure 6.**
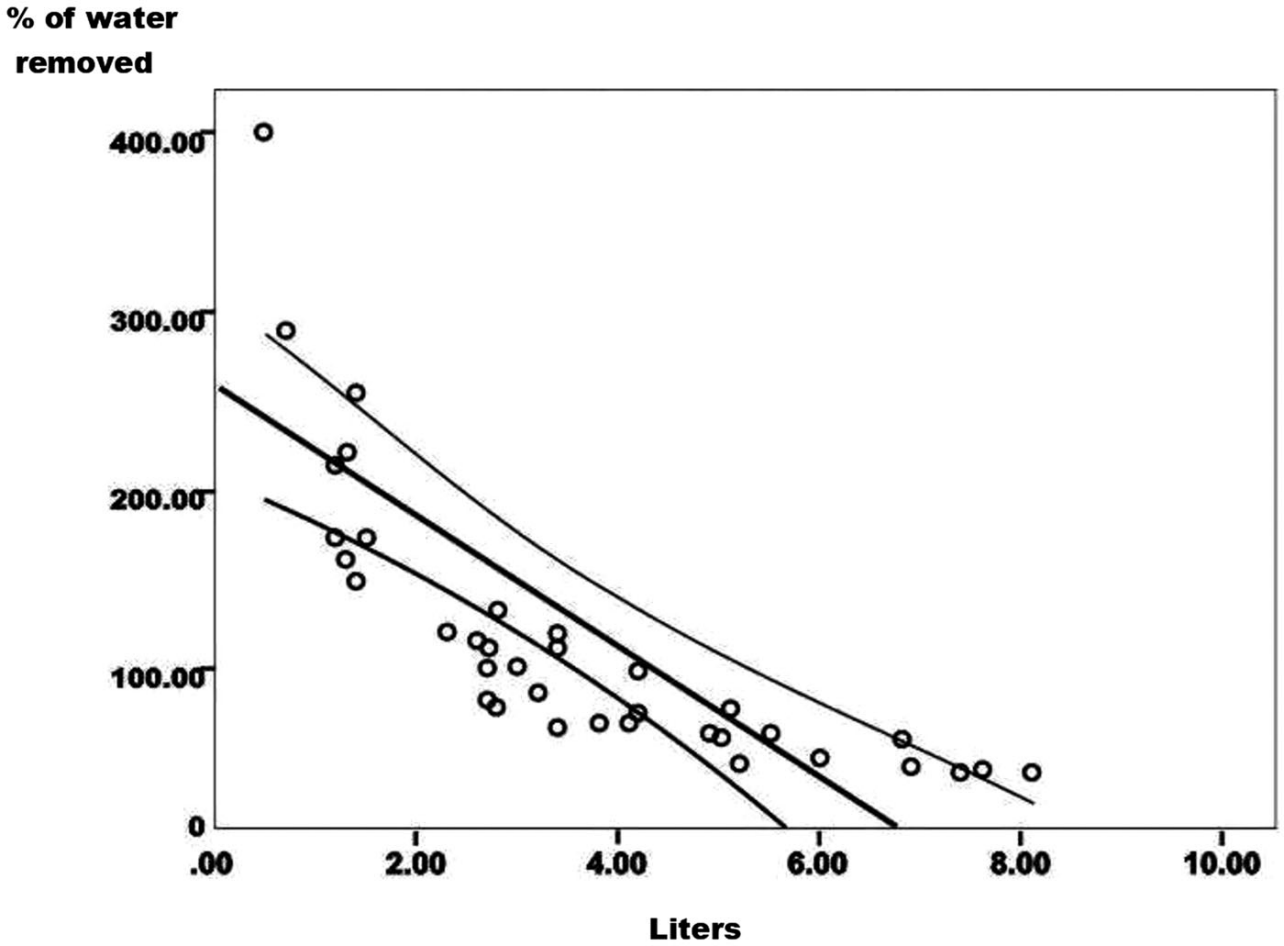
Percent of water removed showed a high inverse correlation, (*r* = -0.70, *p* < 0.0001).The larger the water retained the lower the percent of water removed.

With BIS we estimated further the impact of the UF in the body water distribution. The results obtained after UF showed a significant decrease in TBW Post and ECW Post and at 2 h (*p* < 0.001); a full recovery was observed after 48 and 72 h (Figure 5). In Table 4 and Figure 6, we displayed the results before and after UF. The decrease in BW, TBW; ECW, PV and FO, showed a significant fall after UF, and a significant increase in the PhA (Table 6). We also analyzed these results accordingly to the state of fluid overload. For that purpose, we estimated the amount of FO in liters. We divided the population in two groups the ones with an FO of 2.48 ± 1.24 liters or Low group (*n* = 27); and another with a mean FO of 6.85 ± 1.94 or High group (*n* = 12); the non-paired t test among these groups was significant (*p* < 0.003).

We contrasted the FO in liters against the percent of water removed in liters with UF in the Low and in the High group (Figure 6; *r* = -0.70; *p* < 0.0001); an inverse relationship was clearly shown. The percent of water removed with UF was lower in the High group than in the Low group (Figure 6).

The results in Table 7 shows that the values of total Hb and erythrocyte mass, were similar in the different period studied after UF, and supports that the variations of [Hb] concentration depicted in Figure 3 are secondary to movements of water out of the intravascular space.

**Table 7.** Values of total Hb (THb) and Erythrocyte mass (EM) in the three periods, pre, post and 48/72 h. The differences were non-significant in the three periods.

Periods	THb Pre	THb Post	THb 48/72	EM Pre	EM Post	EM 48/72
*n*	38	38	38	38	38	38
Mean	428 grs	428 grs	429 grs	1.3 L	1.3 L	1.3 L
St Dev	111	110	107	0.34	0.34	0.33

## Discussion

Our results in the large cohort are relevant and showed a significant drop of the SBP, DBP and MAP after UF (Table2). This fall of the BP was more pronounced in the Hypotensive group as is analyzed in Table 3 compared with the Normotensive group. The % PV correlated with the variations of the SBP (Figure 2). The UF in this group had a negative correlation with the PV. The fall in the SBP and MAP showed a low but significant correlation with the UF. In 96 (30%) of the Hypotensive group (Table 3) the SBP mean drop was −40.3 mmHg; 39 of them (49%) had a fall of more than 40 mmHg and 15 of them of more than 60 mmHg with a mean fall of −73 ± 12 mmHg. In total 39 cases of the 321 (12%) had a very severe form of hypotension. The UF was higher and the % PV lower in the Hypotensive group and significantly different from the Normotensive group (Table 3). In conclusion, the UF determines the fall in the PV and the drop in SBP and this effect is more pronounced in the Hypotensive group. In this group, Diabetes occurred in 77% of the patients significantly different from the Normotensive group that was present in 46.4% of the patients. The presence of diabetes, diabetic neuropathy, vasomotor instability in some of these patients may contribute to the tendency to hypotension.

In the group with a SBP >140 mmHg the majority of cases had a SBP between 150 and 240 mm Hg. (Table 4). The fall of the systolic BP was important (-14.7 ± 27.8 mmHg); furthermore 82/200 (40%) patients had a fall of more than 20 mmHg. The occurrence of diabetes and moderate to severe hypertension may be factors of risk to develop hypotension plus, the amount and speed UF and the fall of the PV as was discussed above. In contrast the group <140 mmHg (Table 4), 76% of the patients had a SBP within normal limits 110–140 mmHg, the mean fall of blood pressure was positive 3.34 ± 20.56 mm Hg, and only 13/121 (11.4%) had a SBP drop below 20 mmHg. The patients in this group were more stable during and after HD, even when submitted to large volumes of UF (2.53 ± 0.44 L) and significant falls in % PV (8.4 ± 10.4).

Our studies in the small cohort confirms the findings of Wisemann et al. and Koomans et al. [3–9], that the fall in PV after UF, is one of the variables responsible for the changes in BP and the hemodynamic instability. This suggests that the blood volume regulation, is altered after UF, and may result in complications, such as hypotension [22–24]. In our small cohort, the fall in BW, TBW and ECW after UF supports this contention (Table 6).

We also found that the refill of the PV at two hours was uneven. This irregular refill may be in part the cause of the hypotension and the hemodynamic instability of hemodialysis patients (Figure 4). The refill of the PV was complete in the following 12–24 h, after HD [19,22] as well as the recovery of the, BW, TBW and ECW (Figure 5).

It is important to mention that we analyzed the results of the Bioimpedance (TBW an ECW) liters of volume lost Post Hd and 2 h after and were compared with the Body weight loss, no significant differences were found as is clearly shown in Table 6. The UF in liters was also similar to the fall of BW, TBW, and ECW as shown in the same Table.

In Table 7 we show that the Total Hb and EM values were constant in all periods studied. Suggesting that the changes in Hb concentration (Figure 3) are secondary to movements of plasma water out of the intravascular space, induced by UF.

The changes observed in % PV at two hours after UF is determined by the movement of fluid out of the intravascular space and of the fluid that returns from the interstitial space. By the end of 24 h the PV is back to control values. The results of Bellizzi [7] and Di Lorio [8] on the rapid recovery of the PV 2 h after HD were not confirmed by Koomans et al.[9] or with our results^:^ The fall in the PV persisted for more than two hours in most of our cases (35/38) (Figure 4). Furthermore, the Hb taken at 2 h post HD was not different from the Hb obtained in the Post HD period (Figure 3) (*p=*<0.7). The more plausible explanation for the discrepancy with Bellizzi and Di Lorio [7,8] might be, the clinical conditions of the patients selected for their study, FO, degree of UF, fluid intake in those 2 h and the absence of subjects with malnutrition and edema.

In cases submitted to low UF the recovery of the PV is fast and complete as shown by Wizemann et al. and Koomans et al. [3,4,9] and confirmed with our data in three cases with UF of less than 1.5 liter (Figure 4). Other pertinent observation was, that patients with a low FO, a larger percent of water is extracted after UF [23,24] (Figure 6), and the refill tend to be slower and, in some patients, this may contribute to the appearance of hypotension. In the nine patients with a low FO of 0.5–1.3 L and more than 100-400 percent of water extracted, 3 developed severe SBP hypotension of −30, −34 and −78 mmHg. This group showed a mean drop of −18.44 ± 25.7 mmHg. The patients with a high FO of 5–8.1 L and 25–70% of water extracted only 1/10 patients, with a FO of 5.5 L, had a drop of SBP of −39 mmHg; the mean drop of the SBP in this group was −4.0 ± 15.9 mmHg. The difference between the two means was not significant (*p* = 0.096) even when the difference among groups, was substantial −14.44 mmHg. This data showed a clear tendency to hypotension in the group with a lower FO. Even though the group is small, and the difference was not sufficient to be significant.

The PhA is a measure that estimates the amount of muscular mass. Patients with large FO with or without edema, the fall of the PhA may obliterate the MM gain, and added to FO be a marker of inflammation and increase mortality. [23,25]

The changes of the PV resulting from UF and estimated with Ht, using monitors such as the Crit Line [26–28] probed to be insufficient to assess the real state of the PV. Due to the fact, of the different variables and clinical situations involved, that impact the central blood volume.

This vascular failure to maintain a stable central vascular volume, is secondary not only to the fall in the PV but also to a decrease in the ECW and specially the interstitial fluid [29,30]. The interstitial fluid pressure is negative (-4 mmHg) in normal patients with a low compliance. In contrast, is positive in patients with overhydration and edema (+ 2–3 mmHg or more) with a high interstitial compliance [31]. Only three patients recovered the PV in two hours, when FO range from 500 to 1500 mL. Patients of the High group and higher FO had a higher compliance of the interstitial space; in these cases, the recovery of the PV was slower, and less the percent of fluid extracted with UF, as depicted in Figures 4 and 6.

Once the hemodynamic and neuro-hormonal factors involved in the response to blood volume regulation are stimulated after UF, particularly in diabetic and hypertensive subjects without renal function, the recovery of the BP and the PV depends on the participation of different mechanisms. The amount and speed of fluid loss, the degree of FO, the expansion of the interstitial space, the refill of the PV and the behavior of body fluid dynamics. All of these physiological consequences induced after UF, take hours to days to recover and just, after 48–72 h or less, the HD procedure is repeated, with the same hemodynamic results and probably are responsible for the unpredictable behavior of these patients.

The observation that patients with SBP within normal limits (110–140 mmHg) are more resistant, to the appearance of hemodynamic complications including hypotension, is an important finding to predict the behavior of patients during and after HD. These patients were more hemodynamic stable, had a lower decrease in PV, less FO, lower percent of diabetes and were free of hypotensive drugs.

## Conclusion

These findings suggest that the blood volume regulation in HD patients, with different FO, presence or absence of diabetes and hypertension, is partially lost. The BP behavior after HD and UF may result in several hemodynamic consequences, among them, persistent and significant falls in BP and PV, irregular recovery of the PV that may lead in some cases to severe clinical hypotension.
